# Hyperspectral imaging and machine learning for rapid sensing and visualized retrieval of soil nutrients in high-latitude tea-growing regions

**DOI:** 10.3389/fpls.2026.1842390

**Published:** 2026-06-05

**Authors:** Xuteng Liu, Xiaojia Zhang, Mei Wang, Zhihan Wang, Zhengtong He, Zhiwei Chen, Mengqi Guo, Chunwang Dong

**Affiliations:** 1 Tea Research Institute of Shandong Academy of Agricultural Sciences, Jinan, China; 2 Faculty of Engineering, Architecture and Information Technology, The University of Queensland, Brisbane, QLD, Australia; 3 Shandong Academy of Agricultural Machinery Science, Jinan, China; 4 College of Mechanical and Electronic Engineering, Shihezi University, Shihezi, China

**Keywords:** hyperspectral imaging, machine learning, predictive models, soil nutrients, spatial visualisation

## Abstract

**Introduction:**

Rapid assessment of soil pH and nutrient status in tea plantations is essential for precision fertilisation and ecological management, particularly in high-latitude tea-growing regions where related applications remain insufficiently studied.

**Methods:**

This study developed a rapid, non-destructive framework integrating hyperspectral imaging and machine learning for the detection, retrieval, and spatial visualisation of soil pH, soil organic matter (SOM), alkali-hydrolysable nitrogen (AN), available phosphorus (AP), and available potassium (AK) in the Taishan tea-producing region. A total of 150 soil samples were collected from three profile depths (0–20, 20–40, and 40–60 cm). Hyperspectral images were acquired over 394–1007 nm, and the 481–908 nm range was retained for modelling. Principal component analysis was used to characterise vertical differentiation, while four spectral pre-processing methods, three feature-band selection algorithms—competitive adaptive reweighted sampling (CARS), bootstrapping soft shrinkage (BOSS), and successive projections algorithm (SPA)—and three regression models—partial least squares regression (PLSR), random forest (RF), and support vector regression (SVR)—were systematically compared.

**Results:**

The soils were generally acidic, and SOM, AN, AP, and AK exhibited clear surface enrichment and decreasing trends with increasing depth. Among the feature-selection methods, BOSS showed the best overall performance in reducing spectral redundancy and improving prediction accuracy. The optimal SVR models, combined with parameter-specific pre-processing and BOSS-selected bands, achieved strong predictive performance across all indicators, with prediction-set correlation coefficients (Rp) of 0.94–0.99 and relative percent deviation (RPD) values of 2.963–10.425. Furthermore, pixel-wise reconstruction using threshold masking enabled intuitive two-dimensional visualisation of the spatial distributions of the target soil properties.

**Discussion:**

These results demonstrate that hyperspectral imaging coupled with machine learning provides an effective approach for rapid soil nutrient assessment, spatial visualisation, and digital management in high-latitude tea plantations.

## 1 Introduction

Tea (Camellia sinensis) is one of the world’s most important economic crops, and its growth, development, and tea quality are particularly sensitive to soil environmental conditions (Zhang et al., 2025). The Taishan tea-producing region (36°11′54.36″ N, 117°06′24.34″ E), located on the southern foothills of Mount Tai in Tai’an, Shandong Province, was among the first introduction sites under Shandong Province’s “southern tea introduced to the north” programme and is also one of the major tea-producing areas in northern China (Tang et al., 2023). Soil physicochemical properties are important indicators for evaluating soil fertility status and ecological function, and are of great significance for agricultural production management and the sustainable use of land. Among these, soil pH, soil organic matter (SOM), alkali-hydrolysable nitrogen (AN), available phosphorus (AP), and available potassium (AK) are not only key indicators for assessing soil fertility and ecological function, but also directly influence tea plant growth and nutrient cycling (Verma et al., 2026). Owing to the long-term effects of fertilisation and tea plantation management, the physicochemical properties of tea plantation soils often exhibit marked spatial heterogeneity and vertical differentiation. Under the combined influence of parent material, topography, climate, and anthropogenic management, the soil nutrient status in the Taishan tea-producing region is closely related not only to tea plant growth and tea quality, but also, to some extent, reflects the quality of the regional soil ecological environment. Therefore, rapid monitoring and accurate assessment of these properties are of considerable theoretical and practical significance.

Traditional methods for determining soil physicochemical properties mainly rely on laboratory-based chemical analysis. Although these methods offer high accuracy, they are characterised by complex procedures, long analytical cycles, and relatively high costs, making them unsuitable for rapid large-scale detection (Lu and Zhang, 2026). With the development of spectral sensing technologies, spectroscopic analysis has gradually become an important means of the rapid detection of soil properties. In recent years, the combination of soil spectroscopy and machine-learning algorithms has made significant progress in the prediction of nutrients such as soil organic matter, nitrogen, phosphorus, and potassium (Ribeiro et al., 2026; Correa et al., 2025; Liu M. et al., 2025). Liu et al. (2024) established predictive models for key soil nutrient contents by integrating ultraviolet–visible–near-infrared (UV-Vis-NIR) and mid-infrared (MIR) data with chemometric methods, thereby making approximate quantification of soil nutrient contents possible. Zhang et al. (2024) proposed a partial transfer component regression (PTCR) framework for standard-free calibration transfer of soil near-infrared spectroscopic data. By embedding sample and/or feature selection into transfer component learning and incorporating a small number of target-domain samples, the framework improved the generalisation of soil organic matter prediction models across different regions and instruments. Their results showed that PTCR outperformed conventional transfer-learning approaches, demonstrating its effectiveness in reducing spectral inconsistency and enhancing model transferability. The integration of Visible and Near-Infrared (Vis-NIR) spectroscopy with machine learning has become a global frontier in rapid soil nutrient assessment. Recent international studies have demonstrated its effectiveness not only in laboratory settings but also across multi-scale platforms. For instance, Tagore et al. (2026) highlighted the synergy between Vis-NIR sensors and machine learning models for improving the accuracy of soil property predictions. Furthermore, advancements in airborne hyperspectral imaging (AVIRIS-NG) and its fusion with satellite data (Sentinel-2) have enabled the high-resolution digital mapping of both major soil nutrients and extractable micronutrients across diverse landscapes (Mondal et al., 2025a, b). These global developments underscore the transformative potential of hyperspectral technology in modern precision agriculture. However, several critical research gaps remain unaddressed. First, most current hyperspectral studies on tea plantation soils are concentrated in the traditional acid-soil regions of southern China. High-latitude tea regions, such as the Taishan producing area, possess distinct soil parent materials and experience different leaching intensities and freeze-thaw cycles, resulting in unique spectral response mechanisms that render existing southern-region models potentially inaccurate. Second, while the vertical distribution of nutrients (e.g., across the 0–60 cm profile) is crucial for understanding tea plant root uptake and precision deep-fertilization, most spectral sensing research has focused exclusively on surface soil (0–20 cm), leaving the diagnostic potential of hyperspectral imaging for deep-layer soil properties under-explored. Furthermore, although machine learning has been applied to point-based soil prediction, there is a lack of integrated frameworks that combine advanced feature selection (such as BOSS) with high-resolution pixel-wise reconstruction to visualize the spatial heterogeneity of multiple nutrients simultaneously. Consequently, reports on rapid quantitative detection methods and spatial visualisation retrieval of soil physicochemical properties in the high-latitude Taishan tea-producing region remain scarce. This has constrained precision nutrient management and ecological assessment in tea plantations within this region.

To address these gaps, this study focuses on soils collected from different profile depths (0–60 cm) in the Taishan tea-producing region, with the aim of developing a rapid, non-destructive framework for the detection and spatial visualisation of key soil physicochemical constituents—pH, organic matter, alkali-hydrolysable nitrogen, available phosphorus, and available potassium—based on hyperspectral techniques and machine-learning algorithms. Specifically, the study seeks to: (1) systematically characterise the vertical differentiation patterns of soil physicochemical properties in tea plantation soils and, through principal component analysis (PCA), reveal the intrinsic drivers and coupling mechanisms underlying hyperspectral responses and nutrient enrichment; (2) comprehensively evaluate the effectiveness of four spectral pre-processing strategies and feature-band selection algorithms (CARS, BOSS, and SPA) in spectral dimensionality reduction, noise removal, and key feature enhancement; (3) conduct an in-depth comparison of the predictive accuracy and robustness of linear and non-linear models, including partial least squares regression (PLSR), random forest (RF), and support vector regression (SVR), in order to determine the optimal retrieval architecture for each physicochemical indicator; and (4) achieve high-precision two-dimensional spatial visualisation of soil physicochemical component contents under complex environmental conditions by integrating the optimal predictive model with pixel-level image reconstruction techniques. This study not only overcomes the spatial limitations of traditional chemical analysis, but also aims to provide a strong theoretical foundation and technical support for the precise sensing, digital management, and ecological quality assessment of soil nutrients in high-latitude tea-growing regions.

## 2 Materials and methods

### 2.1 Soil sample preparation

**Figure 1:**
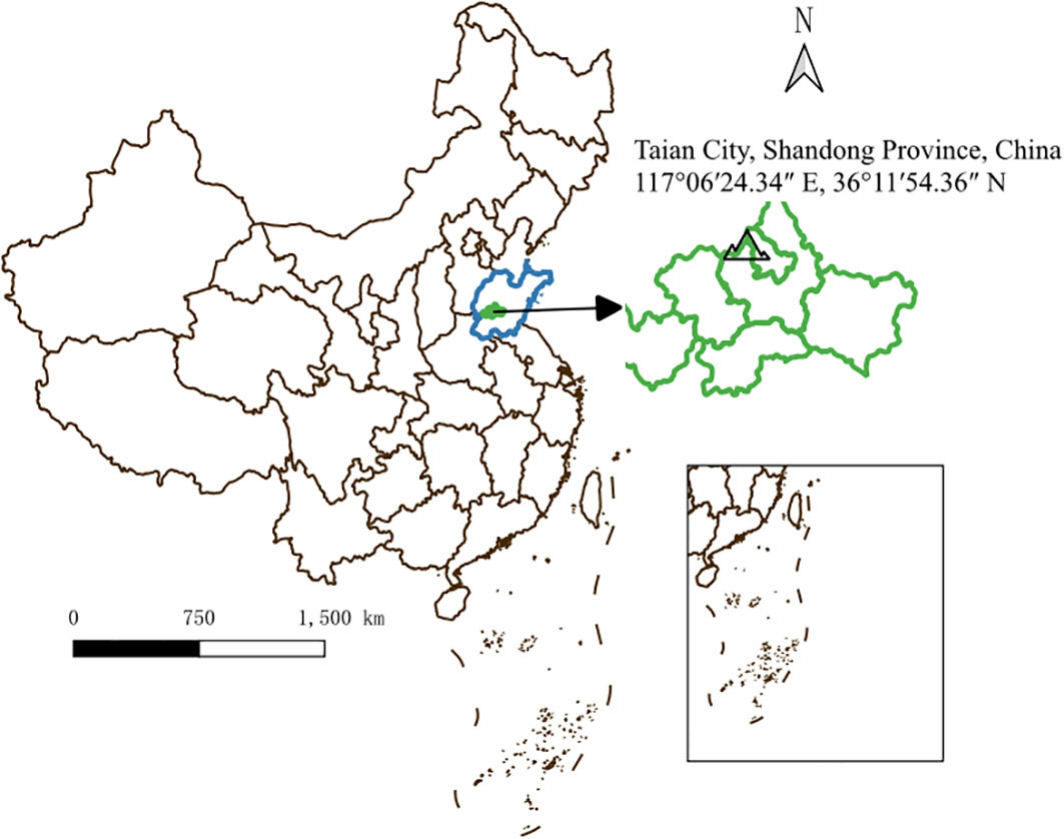
Geographical location of the study area and the distribution of sampling sites. The map indicates the location of the Taishan tea-producing region in Tai’an City, Shandong Province, China, situated at the Wanjishan Experimental and Demonstration Base (117°06′24.34″ E, 36°11′54.36″ N).

The study area is situated at the Wanjishan Experimental and Demonstration Base of the Shandong Institute of Pomology (117°06′24.34″ E, 36°11′54.36″ N) (**Figure 1**). A stratified random, profile-paired sampling strategy was employed for this research: the study area was zoned and stratified, with 50 randomly distributed sampling sites established across the strata. At each site, soil samples were extracted from three depth profiles (0–20 cm, 20–40 cm, and 40–60 cm), forming a “50 × 3” sampling framework that yielded a total of 150 samples. For each layer, five subsamples were collected within a 1–3 m radius of the central site using a quincunx sampling pattern and thoroughly homogenised. A representative composite sample was then taken for subsequent spectral analysis and modelling, aiming to mitigate micro-scale heterogeneity whilst ensuring the spatial representativeness and inter-layer comparability of the samples. Post-extraction, non-soil matter (such as stones, fine tea roots, and straw) was removed without altering the intrinsic composition or particle size of the soil. The samples were then sealed in zip-lock bags, transported to the laboratory, and stored at 4 °C for subsequent physicochemical characterisation and hyperspectral data acquisition.

### 2.2 Determination of key soil physicochemical constituents

Five key soil properties were determined following standard protocols, with the core scientific principles of each method briefly outlined below. Soil pH was measured potentiometrically in a 1:2.5 (m/v) soil-to-water suspension using a calibrated glass electrode, based on the Nernstian response of the electrode to hydrogen ion activity (NY/T 1121.2-2006) (Xia et al., 2026). Soil organic matter (SOM) was quantified via the potassium dichromate–sulphuric acid external heating oxidation–titration method, in which organic carbon is oxidised by excess dichromate under acidic conditions and the remaining oxidant is back-titrated with ferrous ammonium sulphate (NY/T 1121.6-2006) (Bharti et al., 2026). Alkali-hydrolysable nitrogen (AN) was determined by the alkaline hydrolysis diffusion method, whereby organic nitrogen is hydrolysed to ammonia under alkaline conditions at 40 °C for 24 h, the released NH_3_ diffuses into boric acid, and the absorbed ammonia is titrated with standard hydrochloric acid (LY/T 1228-2015) (Zhou et al., 2025). Available phosphorus (AP) was extracted with 0.5 mol L^-^¹ sodium hydrogen carbonate and quantified colorimetrically by the molybdenum-antimony anti-spectrophotometric method at 880 nm, relying on the formation of a phosphomolybdate blue complex (HJ 704-2014) (Jiang et al., 2026). Available potassium (AK) was extracted with 1.0 mol L^-^¹ neutral ammonium acetate and measured by flame photometry, which determines potassium concentration from the emission intensity of K atoms at 766.5 nm (NY/T 889-2004) (Cheng et al., 2025). All analyses included reagent blanks and parallel samples to ensure quality control.

### 2.3 Soil hyperspectral image acquisition

Hyperspectral images of the soil samples were acquired using a push-broom (line-scanning) hyperspectral imaging system (Model: FS-13, Hangzhou CHNSpec Technology Co., Ltd., China). The system operates within the visible and near-infrared (Vis-NIR) spectral range (394–1007 nm), comprising 300 spectral bands with a spectral resolution of 2.5 nm. The hardware platform primarily consists of an FS-13 hyperspectral camera, a halogen illumination source (four 100 W halogen lamps positioned at 45° angles to provide uniform illumination), a motorised linear translation stage, and a computer workstation equipped with data acquisition software. Air-dried and sieved soil samples were spread uniformly in sample dishes, and their surfaces were compacted to reduce roughness. Imaging scans were performed under stable light conditions, with the equipment preheated for 30 minutes prior to acquisition to mitigate baseline drift. The laboratory temperature was maintained at 20 °C, and the humidity was kept at 60%.

Prior to soil spectral scanning, dark current acquisition and white reference calibration were performed. The reflectance was calculated as follows (Guo et al., 2025a), as shown in Equation 1:

(1) $$R\left(\lambda \right)=\frac{I\left(\lambda \right)-I_{d}\left(\lambda \right)}{I_{w}\left(\lambda \right)-I_{d}\left(\lambda \right)}$$

where I is the raw spectral signal of the soil sample, I_d_ is the dark current, and I_w_ represents the white reference response.

A total of 150 hyperspectral images were acquired, and spectral signals were extracted from these images using the Region of Interest (ROI) tool in the ENVI 5.3 software. To mitigate the impacts of sample edge shadows, uneven sample distribution, and localised anomalous pixels, ten ROIs were selected for each soil sample image in accordance with a standardised protocol. These ROIs were preferentially positioned within the central and visually uniform regions of the sample, deliberately avoiding areas characterised by high luminance, shadows, impurities, or anomalous reflectance. The spectral data from the ten ROIs were subsequently averaged to yield a representative spectrum for each sample, which was then utilised for subsequent modelling. This procedure enhanced the stability and robustness of the spectral characterisation, ultimately producing a final dataset comprising 150 hyperspectral records.

### 2.4 Spectral pre-processing

To mitigate noise and scattering effects, and to enhance the predictive modelling potential between the spectra and soil physicochemical properties, this study compared four preprocessing methods applied to the raw reflectance spectra: Savitzky-Golay (S-G) smoothing (Yin et al., 2025), Standard Normal Variate (SNV) transformation (Wang et al., 2025), one-dimensional median filtering (medfilt) (Guo et al., 2025b), and normalize (Liang et al., 2025). Savitzky-Golay filtering employed local polynomial fitting to suppress high-frequency noise whilst preserving spectral shape characteristics. SNV performed row-wise centring and standardisation on each spectrum to attenuate multiplicative scattering and baseline drift induced by particle size, surface roughness, and variations in illumination. Median filtering (medfilt) smoothed the spectral sequence to remove impulse noise and localised outliers. Normalize applied an L2-norm normalize to each spectrum to eliminate the impact of variations in overall reflectance amplitude amongst different samples on the model. Following the preprocessing of the raw spectra, linear Partial Least Squares Regression (PLSR) models were individually established to evaluate and select the optimal preprocessing strategy.

### 2.5 Feature band selection

Following the determination of the optimal preprocessing method, three characteristic waveband selection methods—Competitive Adaptive Reweighted Sampling (CARS) (Luo et al., 2025), Bootstrap of Soft Shrinkage (BOSS) (An et al., 2023), and the Successive Projections Algorithm (SPA) (Liu et al., 2026)—were introduced to further reduce spectral redundancy and collinearity, enhance model interpretability, and improve generalisation ability. CARS iteratively eliminates wavebands with minor contributions through multiple Monte Carlo sampling iterations, employing a’survival of the fittest’ principle. BOSS constructs multiple sub-models via bootstrap resampling and assigns probability weights to wavebands based on their respective contributions across these sub-models. By adopting a ‘soft shrinkage’ strategy, it gradually decreases the selection probability of low-contribution wavebands rather than discarding them outright, thereby suppressing redundancy and noise whilst improving selection stability. SPA selects wavebands with the lowest possible inter-correlation through projection within the variable space, preferentially identifying features with complementary information to minimise redundancy. Finally, PLSR models were individually established for the three combinations comprising the ‘optimal preprocessed spectra + (CARS/BOSS/SPA)’, allowing for a comparative evaluation to determine the optimal waveband selection algorithm.

### 2.6 Model development and performance assessment

Given that the mapping relationship between soil spectra and physicochemical properties is susceptible to the combined effects of factors such as particle size, mineral composition, organic matter, and surface roughness—exhibiting significant non-linearity and regional dependency—relying solely on a linear PLSR model may be insufficient to adequately characterise these complex spectrum-property relationships. To secure the optimal model for estimating soil physicochemical indicators, non-linear Support Vector Regression (SVR) (Dong et al., 2021) and Random Forest (RF) (Jiang et al., 2025) models were independently constructed for comparative evaluation. Random Forest (RF) operates as an ensemble learning technique that aggregates the outputs of multiple decision trees to enhance predictive stability and mitigate over-fitting. In this study, the number of decision trees (Ntree) was optimized through a grid search ranging from 25 to 1000 with an increment of 25, where the optimal Ntree was determined by minimizing the Root Mean Square Error of Calibration (RMSEC). Complementarily, Support Vector Regression (SVR) utilizes kernel functions to map input variables into a high-dimensional feature space. The SVR model was implemented with integrated feature scaling for both predictors and target variables to ensure numerical stability. The critical hyperparameters, including the penalty factor (*ϵ*) and the kernel parameter (*γ*), were optimized through an automated searching process to minimize the structural risk and prediction error. This kernel-based methodology is particularly effective for addressing the non-linearities and small-sample constraints frequently encountered in soil spectroscopy. Prior to model development, the Kennard-Stone (K-S) algorithm (Liu et al., 2024) was employed to partition the dataset. The 150 samples were divided into a calibration set and a prediction set at a ratio of 7:3, comprising 105 and 45 samples, respectively. The correlation coefficient of calibration (Rc), correlation coefficient of prediction (Rp), root mean square error of calibration (RMSEC), root mean square error of prediction (RMSEP), and relative percent deviation (RPD) were adopted as the final performance metrics to evaluate the established models, as shown in Equations 2–6.

(2) $$R_{c}=\frac{\sum_{i=1}^{n}\left(y_{i}-\bar{y}\right)\left(y_{i}^{\prime }-{\bar{y}}^{\prime }\right)}{\sqrt{\sum_{i=1}^{n}{\left(y_{i}-\bar{y}\right)}^{2}\sum_{i=1}^{n}{\left(y_{i}^{\prime }-{\bar{y}}^{\prime }\right)}^{2}}}$$

(3) $$R_{p}=\frac{\sum_{i=1}^{m}\left(y_{i}-\bar{y}\right)\left(y_{i}^{\prime }-{\bar{y}}^{\prime }\right)}{\sqrt{\sum_{i=1}^{m}{\left(y_{i}-\bar{y}\right)}^{2}\sum_{i=1}^{m}{\left(y_{i}^{\prime }-{\bar{y}}^{\prime }\right)}^{2}}}$$

(4) $$RMSEC=\sqrt{\frac{\sum_{i=1}^{n_{c}}{\left(y_{i}-\hat{y_{i}}\right)}^{2}}{n_{c}}}$$

(5) $$RMSEP=\sqrt{\frac{\sum_{i=1}^{n_{p}}{\left(y_{i}-\hat{y_{i}}\right)}^{2}}{n_{p}}}$$

(6) $$RPD=\frac{SD_{p}}{RMSEP}$$

Where the parameters are defined as follows:

*y_i_* represents the measured value of the *i*-th soil sample.

$y_{i}^{\prime }$ and $\hat{y_{i}}$ represent the predicted value of the *i*-th soil sample.

$\bar{y}$ and ${\bar{y}}^{\prime }$ are the mean values of the measured and predicted   results, respectively.

*n* and *n_c_* denote the number of samples in the calibration set (*n*   = *n_c_* = 105).

m and *n_p_* denote the number of samples in the prediction set (*m*   = *n_p_* = 45).

*SD_p_* is the standard deviation of the measured values in the prediction set.

### 2.7 Visualisation of soil physicochemical component contents

Based on the optimal regression models established for the soil physicochemical components, pixel-wise spatial retrieval and visualisation of the hyperspectral images were achieved. Initially, the calibrated hyperspectral images were imported. The spectral bands of the 3D hyperspectral image cubes (921 × 480 × 300) were trimmed, retaining the 481–908 nm range (211 spectral bands). Subsequently, the spatial dimensions were unfolded into a 2D matrix (442080 × 211), and a binary mask was generated via thresholding to eliminate background regions. Following this, the optimal preprocessing and waveband selection techniques specific to each physicochemical component were applied. After dimensionality reduction via Principal Component Analysis (PCA), the data were fed into the fully trained optimal regression models to generate predicted values. These predictions were subsequently back-filled according to the mask indices and reconstructed into a 2D spatial distribution matrix. Finally, image colour rendering was performed. The 2nd and 98th percentiles of the valid predicted values were extracted as the lower and upper limits for colour map stretching, respectively. The Turbo pseudo-colour palette was then applied to illustrate the spatial gradient distribution of each physicochemical component’s concentration.

### 2.8 Data analysis software

The prediction models for the soil physicochemical components in this study were all developed using MATLAB 2021a (The MathWorks Inc., 2021); the soil hyperspectral data were extracted using ENVI 5.6 software (NV5 Geospatial, 2020); and all figures were generated using Origin 2025 (OriginLab Corp., 2024).

## 3 Results and analysis

### 3.1 Analysis of soil physicochemical properties

**Figure 2:**
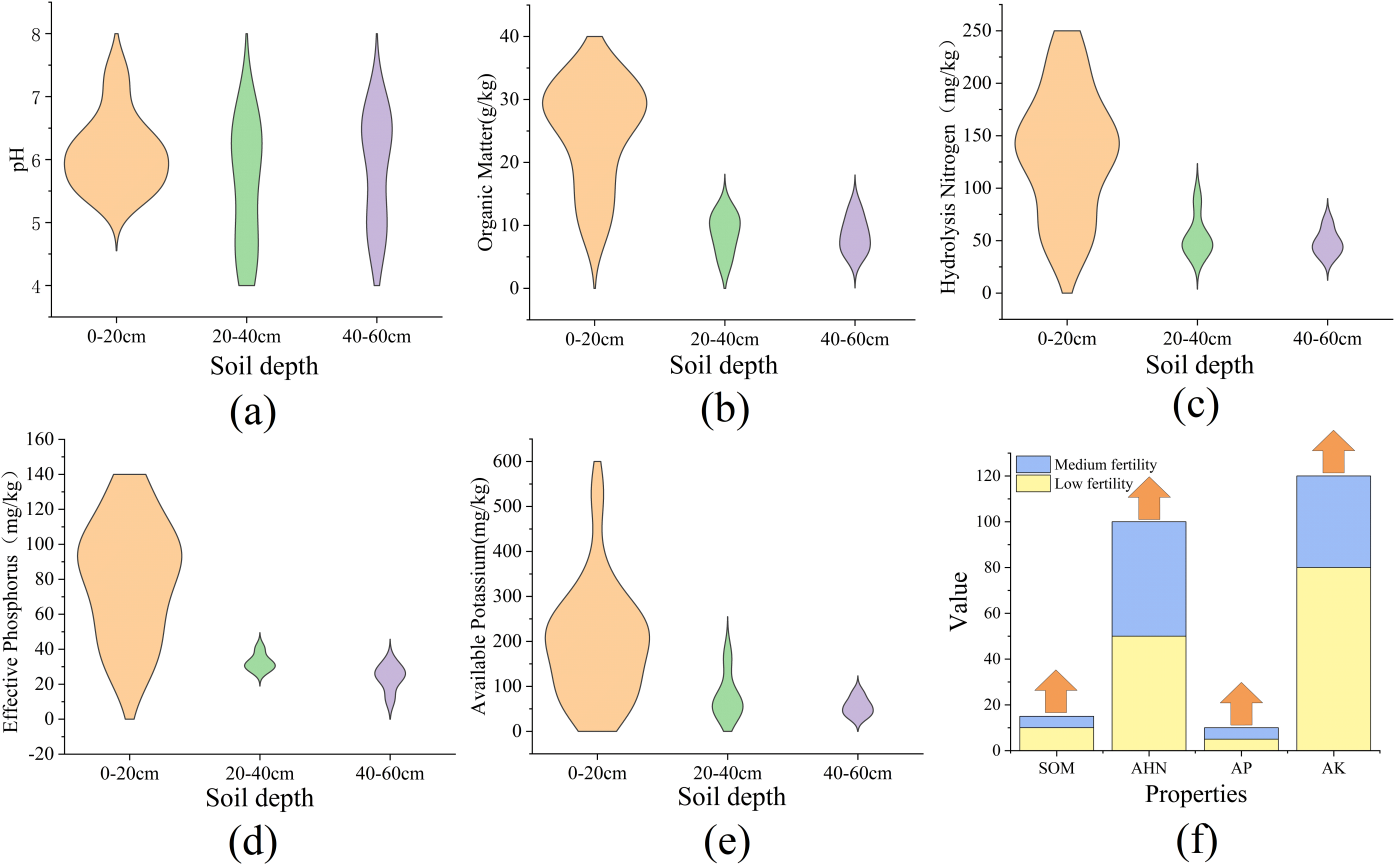
Contents of physicochemical components and soil fertility at different soil depths in the Tai’an tea-growing area. **(a)** pH; **(b)** Soil organic matter; **(c)** Alkali-hydrolyzable nitrogen; **(d)** Available phosphorus; **(e)** Available potassium; **(f)** Fertility evaluation criteria for different physicochemical components.

The descriptive statistics of the physicochemical properties across different soil depths in the Tai’an tea garden are summarised in **Table 1**. To provide a more intuitive visualisation of the data distribution, violin plots were generated (**Figure 2**). Overall, the tea garden soils exhibit acidic characteristics (pH< 7). The mean pH values for the different soil layers are 6.21 (0–20 cm), 5.58 (20–40 cm), and 5.88 (40–60 cm), respectively. The surface soil exhibits relatively mild acidity, whereas the mid- and deep-layer soils display stronger and comparable levels of acidity. As illustrated in **Figure 2a**, the pH distribution of the surface soil is relatively concentrated. Conversely, the distribution range broadens significantly in the deeper layers, accompanied by an increase in acidity. This indicates that the physicochemical environment of the deeper soils is more profoundly influenced by the parent material and leaching processes. Generally, the various soil physicochemical indicators (SOM, AN, AP, and AK) demonstrate a pronounced vertical gradient distribution along the soil profile; specifically, they tend to accumulate in the surface layer and progressively decline with increasing soil depth. Furthermore, the degree of spatial variability differs significantly amongst the various indicators. In a two-year field experiment implementing varying phosphorus source conditions, Guo et al. (2024) observed that soil nutrient concentrations consistently exhibited a decreasing trend with increasing depth across different time periods. This distribution pattern indicates that soil nutrient dynamics within the tea garden are primarily governed by the combined effects of fertilisation management, litter return, root activity, and the leaching and migration of nutrients.

**Table 1:** Soil physicochemical properties of tea plantation soils in Tai’an.

Properties	Depth(cm)	Min	Max	Median	Mean	STD	CV
pH	0-20	5.45	7.47	6.16	6.21	0.59	0.09
	20-40	4.44	6.50	5.91	5.58	0.99	0.18
	40-60	4.83	6.55	6.45	5.88	0.78	0.13
SOM (g/kg)	0-20	9.19	34.96	27.14	24.25	7.48	0.31
	20-40	4.06	11.70	9.20	8.73	2.87	0.33
	40-60	5.83	12.61	8.38	8.53	2.52	0.30
AN (mg/kg)	0-20	43.68	239.87	132.81	128.06	56.02	0.44
	20-40	42.00	88.28	46.90	54.29	17.24	0.32
	40-60	35.56	67.13	47.36	48.28	10.88	0.23
AP (mg/kg)	0-20	9.28	137.05	74.63	72.49	36.09	0.50
	20-40	28.40	40.49	31.75	32.64	4.32	0.13
	40-60	11.84	31.75	24.68	23.79	6.60	0.28
AK (mg/kg)	0-20	37.00	531.00	169.00	180.50	116.43	0.65
	20-40	45.00	164.00	58.00	77.60	44.41	0.57
	40-60	36.00	85.00	55.00	55.00	18.08	0.33

Min: minimum; Max: maximum; SD:standard deviation; CV: coefficient of variation; SOM: soil organic matter; AN: alkali-hydrolyzable nitrogen; AP: available phosphorus; AK: available potassium.

### 3.2 PCA clustering and correlation heatmap analysis of soil hyperspectra and physicochemical properties

**Figure 3:**
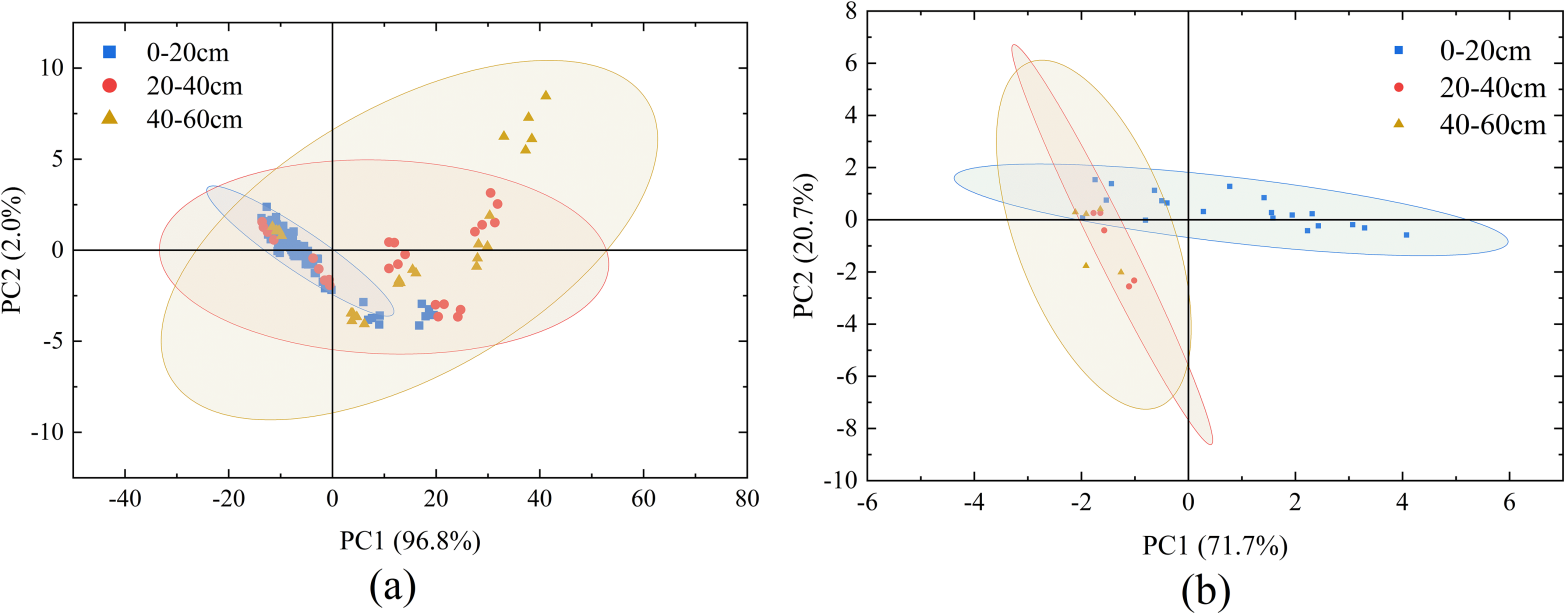
PCA clustering plot of soil hyperspectral data and physicochemical properties. **(a)** Hyperspectral data; **(b)** Physicochemical properties.

To elucidate the spatial differentiation and underlying driving mechanisms of the soil hyperspectral characteristics and physicochemical properties across varying depths (0–20 cm, 20–40 cm, and 40–60 cm), Principal Component Analysis (PCA) was performed independently on the spectral reflectance and the physicochemical indicators (pH, SOM, AN, AP, and AK). The results are illustrated in **Figure 3**.

#### 3.2.1 Vertical differentiation patterns of soil hyperspectral characteristics across soil layers

The PCA results for the hyperspectral data (**Figure 3a**) indicate that the first two principal components cumulatively explained 98.8% of the total variance (PC1: 96.8%, PC2: 2.0%), demonstrating that these two components can effectively characterise the primary variations in the soil hyperspectral information. Within the principal component space, the soil samples exhibit a pronounced gradient distribution pattern along the profile depth. The surface soil (0–20 cm) clusters tightly along the negative semi-axis of PC1, forming a narrow and concentrated confidence ellipse, which indicates a high degree of consistency in its spectral characteristics. In contrast, the mid- and deep-layer soil samples display a substantial spread along PC1 (primarily extending towards the positive semi-axis), and their respective confidence ellipses overlap considerably. This indicates that as depth increases, the spatial heterogeneity of the soil’s spectral features is significantly enhanced, whilst the mid- and deep-layer soils demonstrate a high degree of similarity in their optical responses.

#### 3.2.2 Dominant factors and clustering mechanisms of major soil physicochemical components

The PCA results for the physicochemical properties (**Figure 3b**) further elucidate the underlying biochemical and physical nature of the aforementioned spectral stratification. PC1 and PC2 of the physicochemical properties accounted for 71.7% and 20.7% of the total variance, respectively. An analysis of the variable loadings demonstrates that soil organic matter (SOM), alkali-hydrolysable nitrogen (AN), available phosphorus (AP), and available potassium (AK) all exhibit exceptionally high positive loadings on PC1 (0.480–0.509); consequently, PC1 characterises a’nutrient and organic matter enrichment factor’. Conversely, pH possesses an absolutely dominant positive loading on PC2 (0.928) whilst displaying an extremely weak loading on PC1 (-0.162), indicating that PC2 primarily characterises an’acidity-alkalinity factor’ that is independent of the nutrient gradient. Within the PCA space, the surface (0–20 cm) samples are predominantly distributed across the high-value region of PC1, whereas the mid- and deep-layer samples are strictly confined to the negative-value region of PC1. This confirms that with increasing soil depth—in the absence of anthropogenic disturbances and surface material inputs—the mid- and deep-layer soils exhibit general nutrient impoverishment and homogenisation. This finding corresponds seamlessly with the conclusions drawn in Section 3.1 regarding the distribution of soil physicochemical component concentrations.

#### 3.2.3 Correlation analysis of soil physicochemical components

**Figure 4:**
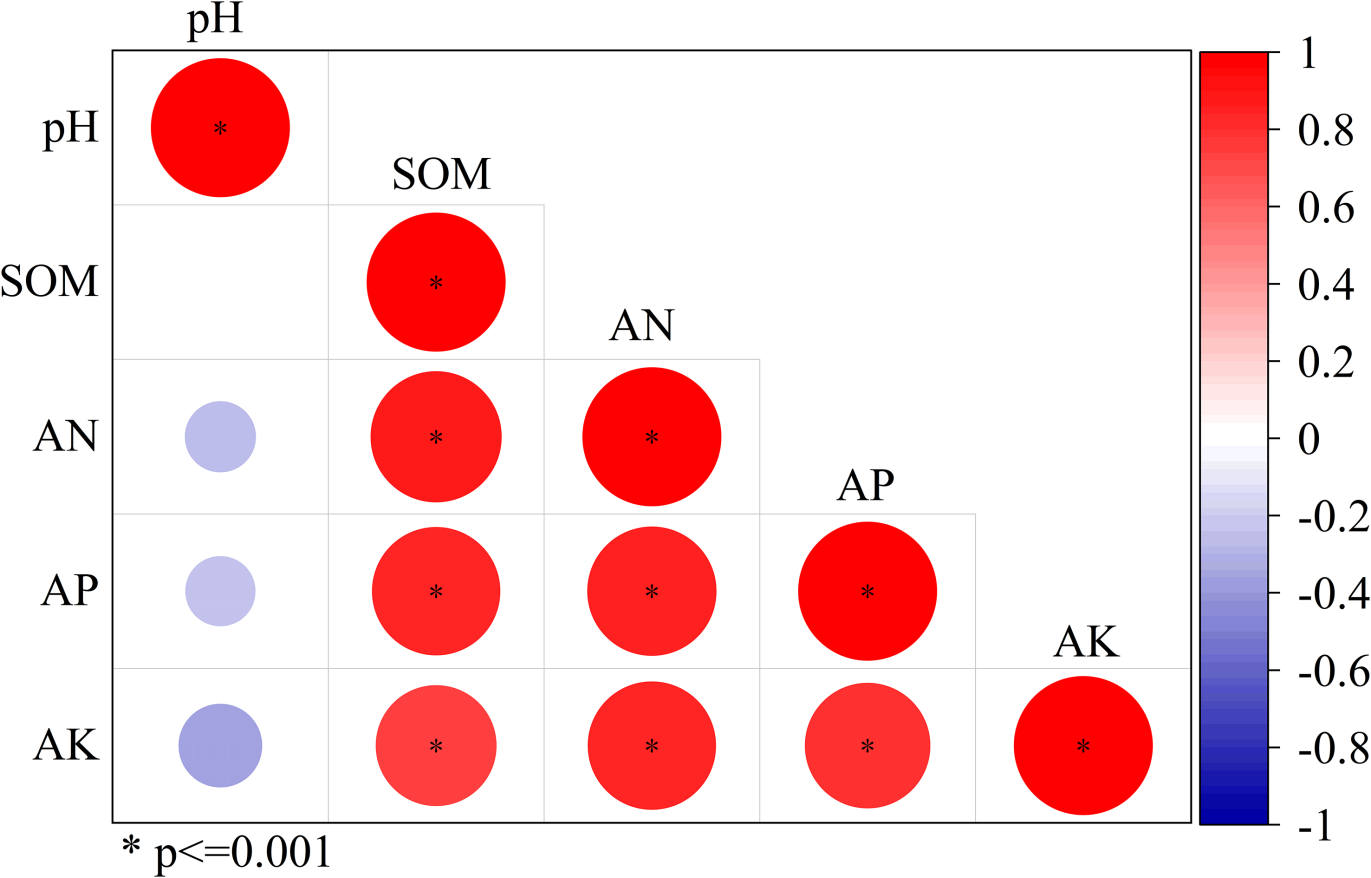
Correlation heatmap of soil physicochemical properties.

Prior to conducting the correlation analysis, a Shapiro-Wilk test was initially employed to assess the normality of the soil physicochemical indicators. The results indicated that certain physicochemical indicators in this study (such as organic matter, alkali-hydrolysable nitrogen, available phosphorus, and available potassium) did not satisfy the assumption of a normal distribution. Consequently, Spearman’s rank correlation analysis was adopted to evaluate the relationships amongst the various soil physicochemical properties, with the results visually presented via a correlation heatmap (**Figure 4**). The findings reveal a distinct positive correlation between soil organic matter (SOM) and the primary nutrient indicators: alkali-hydrolysable nitrogen (AN), available phosphorus (AP), and available potassium (AK). This demonstrates the pivotal role of soil organic matter in nutrient cycling and supply, whilst also indicating that nutrient elements such as nitrogen, phosphorus, and potassium exhibit strong synergistic variations within the soil. Furthermore, strong positive correlations were similarly observed amongst AN, AP, and AK, further reflecting the coupled variation patterns of different nutrient elements in the tea garden soils. In contrast, soil pH was negatively correlated with AN, AP, and AK, whilst its correlation with SOM was relatively weak, suggesting a certain divergence between variations in soil acidity/alkalinity and the nutrient enrichment process. Overall, the correlation analysis demonstrates that pronounced synergistic relationships exist amongst the soil fertility indicators in the tea garden, whereas pH exhibits relatively independent variation characteristics. This is in excellent agreement with the aforementioned nutrient distribution patterns along the soil profile.

### 3.3 Hyperspectral analysis of soil and optimisation of spectral pre-processing

#### 3.3.1 Functional group analysis of soil hyperspectra

**Figure 5:**
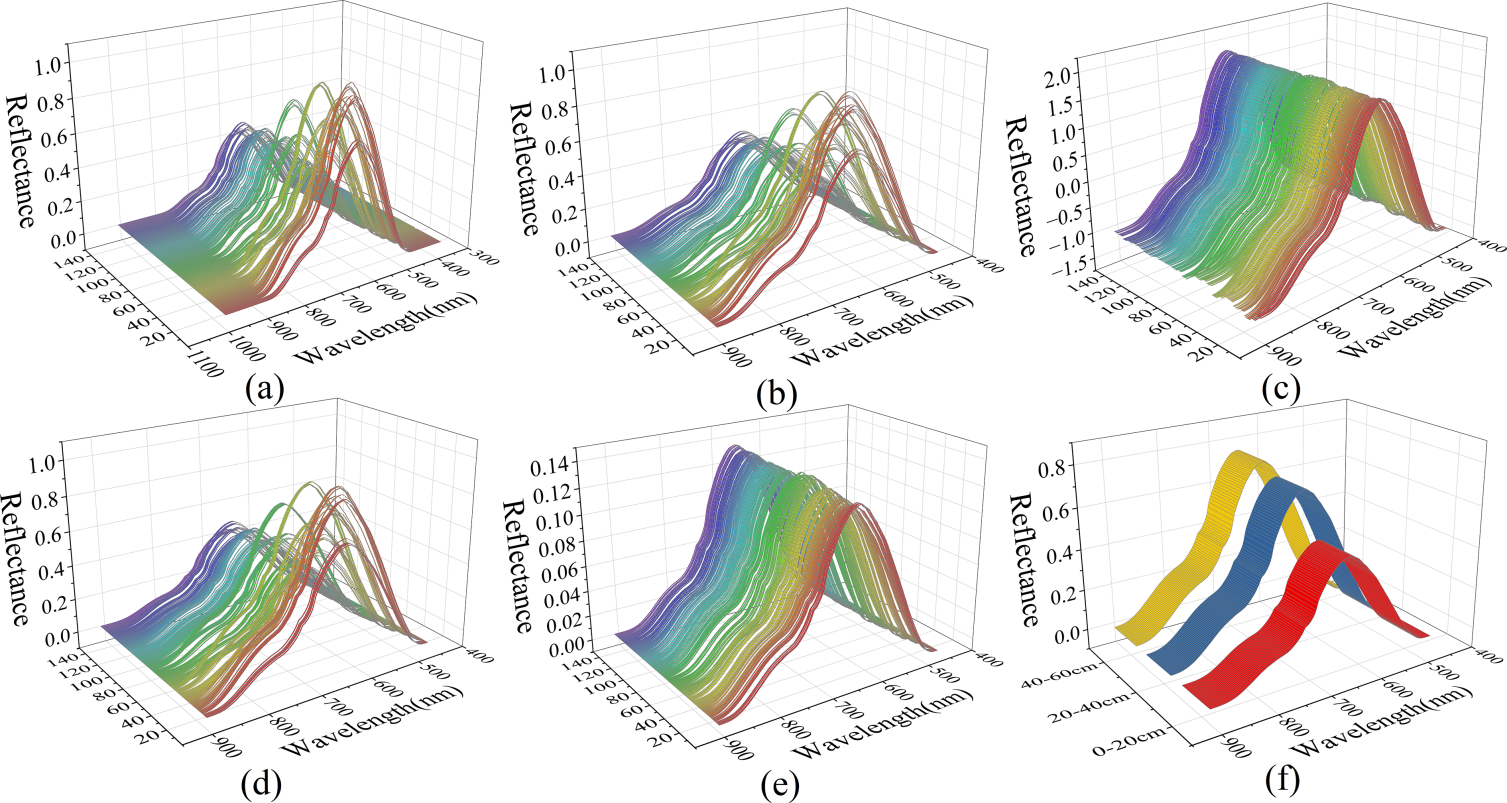
Soil hyperspectral reflectance curves under different preprocessing methods. **(a)** Raw spectra; **(b)** Savitzky-Golay (S-G) smoothing; **(c)** standard normal variate (SNV) transformation; **(d)** median filtering (Medfilt); **(e)** normalization (Normalize); and **(f)** mean spectral reflectance of soils at different depths (0–20 cm, 20–40 cm, and 40–60 cm).

The raw spectral curves of the soil samples were plotted (**Figure 5a**). Overall, the various soil samples exhibited relatively consistent spectral morphological characteristics within the 480–900 nm waveband range. In the visible light region (400–700 nm), the soil spectra are primarily governed by the electron transition absorption of iron oxides. Specifically, the absorption feature near 520 nm is typically associated with the crystal field electron transitions of Fe^3+^ in goethite and hematite, whilst the broad absorption band around 650–700 nm is similarly closely related to the electron transition processes of iron oxides. Collectively, these absorption features determine the variations in soil colour and reflectance (Viscarra Rossel et al., 2006; Ben-Dor et al., 2009). As the wavelength extends into the near-infrared region (700–900 nm), the spectra gradually transition from being dominated by electron transition absorption to being controlled by molecular vibration absorption. Spectral variations in this region are primarily associated with the higher-order overtone vibrations of groups such as O-H and C-H, as well as soil moisture absorption; concurrently, they are influenced by the scattering effects of soil particles. **Figure 5f** further illustrates the variation characteristics of the average spectral reflectance across different soil layers. The results indicate that the surface soil (0–20 cm) exhibits the lowest overall reflectance, whereas the reflectance gradually increases in the 20–40 cm and 40–60 cm layers. This trend is fundamentally consistent with the vertical distribution characteristics of the soil physicochemical properties. The organic matter content in the surface soil is significantly higher than that in the mid- and deep-layer soils, and the strong light-absorbing properties of humus markedly reduce the soil spectral reflectance. Simultaneously, nutrient indicators such as alkali-hydrolysable nitrogen, available phosphorus, and available potassium also demonstrate higher concentrations in the surface soil and progressively decrease with increasing soil depth, indicating a pronounced surface enrichment characteristic of soil nutrients along the profile. With increasing soil depth, the organic matter and nutrient contents decrease, and the scattering effect of mineral particles gradually intensifies, thereby leading to an overall elevation in soil reflectance. Therefore, the vertical variation in soil spectral reflectance is not only governed by the organic matter content but is also closely associated with the synergistic variations of nutrients such as nitrogen, phosphorus, and potassium. This demonstrates that the nutrient distribution characteristics of the soil profile can be distinctly reflected in the hyperspectral reflectance.

#### 3.3.2 Optimisation of spectral pre-processing

To minimise the influence of instrumental noise, baseline drift, and soil particle scattering effects on the spectral signals, this study employed S-G, SNV, Medfilt, and normalize, respectively, to pre-process the spectral data (Figures. 5b–5e). Prior to establishing the partial least squares regression (PLSR) models, principal component analysis (PCA) was first employed to reduce the dimensionality of the high-dimensional spectral data. The optimal number of principal components (PCs) was determined based on cross-validation results to mitigate multicollinearity amongst the spectral variables and prevent model overfitting. Given that the 394–481 nm and 908–1007 nm ranges predominantly consisted of noise, these spectral regions were eliminated prior to modelling. The 481–908 nm range (comprising 211 bands in total) was retained for model development. The optimal number of PCs and the model evaluation metrics for each physicochemical parameter under the different pre-processing methods are detailed in **Table 2**.

**Table 2:** Performance of PLSR models for predicting soil physicochemical properties under different spectral preprocessing methods.

Properties	Preprocessing	PCs	Rc	RMSEC	Rp	RMSEP	RPD
pH	Raw	12	0.78	0.46	0.71	0.47	1.219
	S-G	8	0.67	0.55	0.74	0.43	1.075
	SNV	11	0.73	0.48	0.71	0.50	1.079
	**Medfilt**	**13**	**0.76**	**0.46**	**0.85**	**0.41**	**1.416**
	Normalize	12	0.71	0.49	0.83	0.42	1.311
SOM	Raw	10	0.92	3.52	0.93	3.80	2.466
	S-G	10	0.93	3.43	0.93	3.75	2.535
	SNV	9	0.93	3.66	0.92	3.84	2.592
	**Medfilt**	**12**	**0.95**	**3.07**	**0.95**	**3.04**	**3.118**
	Normalize	9	0.92	3.71	0.94	3.50	2.815
AN	Raw	11	0.82	31.77	0.80	38.71	1.330
	S-G	9	0.80	32.61	0.79	40.07	1.282
	SNV	10	0.82	33.91	0.80	34.38	1.498
	Medfilt	12	0.85	29.17	0.85	36.13	1.429
	**Normalize**	**10**	**0.82**	**33.66**	**0.84**	**33.01**	**1.536**
AP	Raw	10	0.75	23.25	0.79	23.93	1.330
	S-G	10	0.76	22.77	0.79	23.94	1.360
	SNV	10	0.73	23.33	0.73	28.22	1.030
	**Medfilt**	**13**	**0.79**	**22.70**	**0.79**	**22.09**	**1.397**
	Normalize	10	0.73	23.80	0.79	24.46	1.221
AK	Raw	11	0.80	54.24	0.81	70.16	1.212
	S-G	10	0.80	54.13	0.80	71.26	1.214
	SNV	10	0.77	65.96	0.81	56.47	1.372
	**Medfilt**	**14**	**0.82**	**53.82**	**0.84**	**67.31**	**1.481**
	Normalize	10	0.78	61.58	0.83	59.87	1.386

Bold values indicate the optimal preprocessing method for each indicator.

The model evaluation results indicate that the responses of the various physicochemical parameters to the spectral pre-processing methods differ significantly. Notably, the soil organic matter (SOM) model demonstrated the highest prediction accuracy; under the Medfilt pre-processing condition, the PLSR model achieved optimal predictive performance (PCs = 12, Rc = 0.95, Rp = 0.95), with a ratio of performance to deviation (RPD) reaching 3.118. The application of different pre-processing methods was found to enhance model performance to varying degrees. For the AN model, normalize pre-processing yielded an RPD value of 1.536, whilst Medfilt pre-processing achieved comparatively superior predictive results for the pH, AK, and AP models. Taking all model evaluation metrics into consideration, this study established the optimal spectral pre-processing strategies for the respective physicochemical parameters: Medfilt pre-processing for SOM, pH, AP, and AK, and normalize pre-processing for AN. These findings suggest that appropriate spectral pre-processing can significantly improve model stability and enhance the extraction of spectral feature information.

### 3.4 Selection of characteristic bands from soil hyperspectra

**Figure 6:**
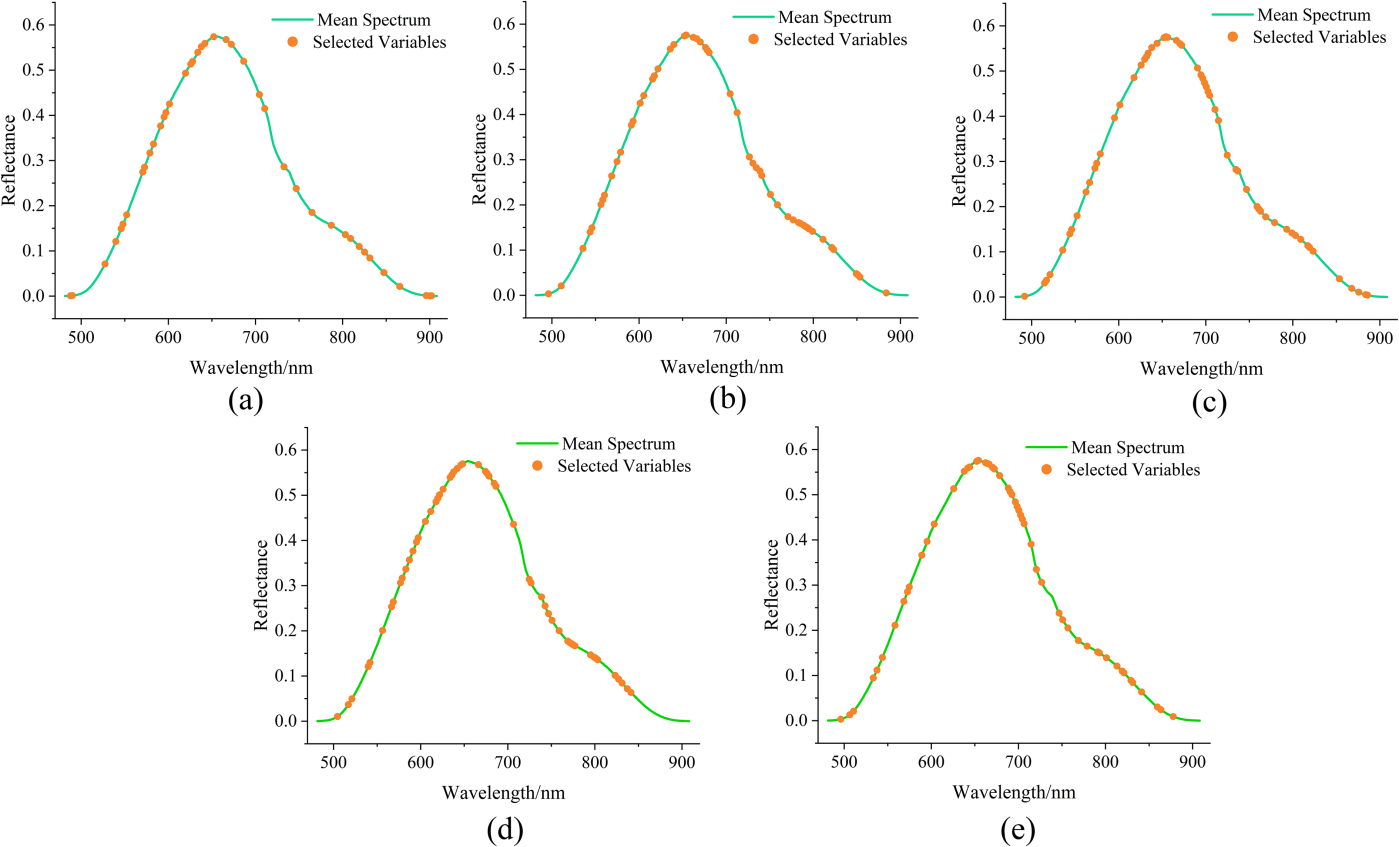
Distribution of characteristic wavelengths selected by the optimal variable selection method (BOSS) for different soil physicochemical properties based on the mean soil spectral curve. **(a)** pH; **(b)** SOM; **(c)** AN; **(d)** AP; and **(e)** AK.

To reduce redundant information in the hyperspectral data and improve the predictive capacity of the models, three variable selection methods—CARS, BOSS, and SPA—were employed to optimise the spectral variables following optimal spectral pre-processing. These were then combined with partial least squares regression (PLSR) models to conduct predictive analyses of the various soil physicochemical properties (**Table 3**). Overall, whilst significantly reducing the number of spectral variables, the variable selection methods exerted varying degrees of impact on the predictive performance of the models. For the soil pH prediction model, under the Medfilt pre-processing condition, CARS, BOSS, and SPA selected 34, 36, and 73 characteristic bands, respectively, representing a substantial reduction in variable quantity compared to the original model. Amongst these, BOSS yielded the lowest RMSEC (0.40) and achieved an Rc of 0.82, a marginal improvement over the original model. This indicates that robust predictive stability was maintained even with a significant reduction in variable dimensionality. For soil organic matter (SOM), the enhancement in model accuracy achieved through variable selection was relatively pronounced. Under the Medfilt pre-processing condition, the BOSS method selected 53 characteristic bands, maximising the predictive accuracy of the model (Rp = 0.96, RPD = 3.405) and outperforming the model utilising all bands. Regarding available hydrolysable nitrogen (AN), under the normalize pre-processing condition, variable selection similarly improved the predictive accuracy of the model. The BOSS method selected 58 characteristic bands, elevating Rp to 0.89 and achieving an RPD of 1.938, which was markedly superior to the original model. For soil available phosphorus (AP), after the BOSS method selected 57 characteristic bands, the model’s predictive accuracy reached Rp = 0.78 and RPD = 1.424, surpassing both the original model and the other variable selection methods. For soil available potassium (AK), BOSS selected 55 characteristic bands, yielding a predictive accuracy (Rp = 0.86, RPD = 1.870) superior to that of the original model. In contrast, whilst SPA selected the fewest variables (8), the predictive performance of the model declined considerably, indicating that an excessive reduction in the number of variables can diminish the model’s capacity to represent spectral information. The characteristic bands selected by the optimal band selection algorithms for the prediction models of each physicochemical component are illustrated in **Figure 6**. In summary, characteristic band selection not only effectively reduced the dimensionality of the spectral variables but also enhanced the predictive performance of the models to a certain degree. The key spectral variables obtained through this screening provide crucial input features for the subsequent construction of machine learning models, facilitating further improvements in the predictive accuracy of soil physicochemical properties.

**Table 3:** Performance comparison of PLSR models for predicting soil physicochemical properties based on different spectral variable selection methods.

Properties	Preprocessing	Method	Variable number	PCs	Rc	RMSEC	Rp	RMSEP	RPD
pH	Medfilt	Raw	211	13	0.76	0.46	0.85	0.41	1.416
		CARS	34	10	0.76	0.45	0.85	0.42	1.318
		BOSS	**36**	**13**	**0.82**	**0.40**	**0.90**	**0.35**	**1.858**
		SPA	73	14	0.74	0.47	0.83	0.44	1.349
SOM	Medfilt	Raw	211	12	0.95	3.07	0.95	3.04	3.118
		CARS	44	11	0.93	3.53	0.94	3.44	2.914
		BOSS	**53**	**11**	**0.95**	**3.02**	**0.96**	**2.84**	**3.405**
		SPA	45	9	0.90	4.11	0.92	4.15	2.256
AN	Normalize	Raw	211	10	0.82	33.66	0.84	33.01	1.536
		CARS	23	10	0.84	30.66	0.90	28.34	1.882
		BOSS	**58**	**11**	**0.83**	**33.27**	**0.89**	**26.15**	**1.938**
		SPA	28	7	0.80	35.12	0.76	38.81	1.283
AP	Medfilt	Raw	211	13	0.79	22.70	0.79	22.09	1.397
		CARS	34	10	0.75	24.34	0.75	23.53	1.308
		BOSS	**57**	**10**	**0.81**	**21.69**	**0.78**	**22.47**	**1.424**
		SPA	69	9	0.74	23.86	0.77	25.65	1.161
AK	Medfilt	Raw	211	14	0.82	53.82	0.84	67.31	1.481
		CARS	30	8	0.78	68.56	0.81	69.61	1.298
		BOSS	**55**	**10**	**0.77**	**76.44**	**0.86**	**50.54**	**1.870**
		SPA	8	6	0.59	82.13	0.69	96.48	0.764

Bold values indicate the optimal preprocessing method for each indicator.

### 3.5 Optimisation of prediction models for soil physicochemical components

**Figure 7:**
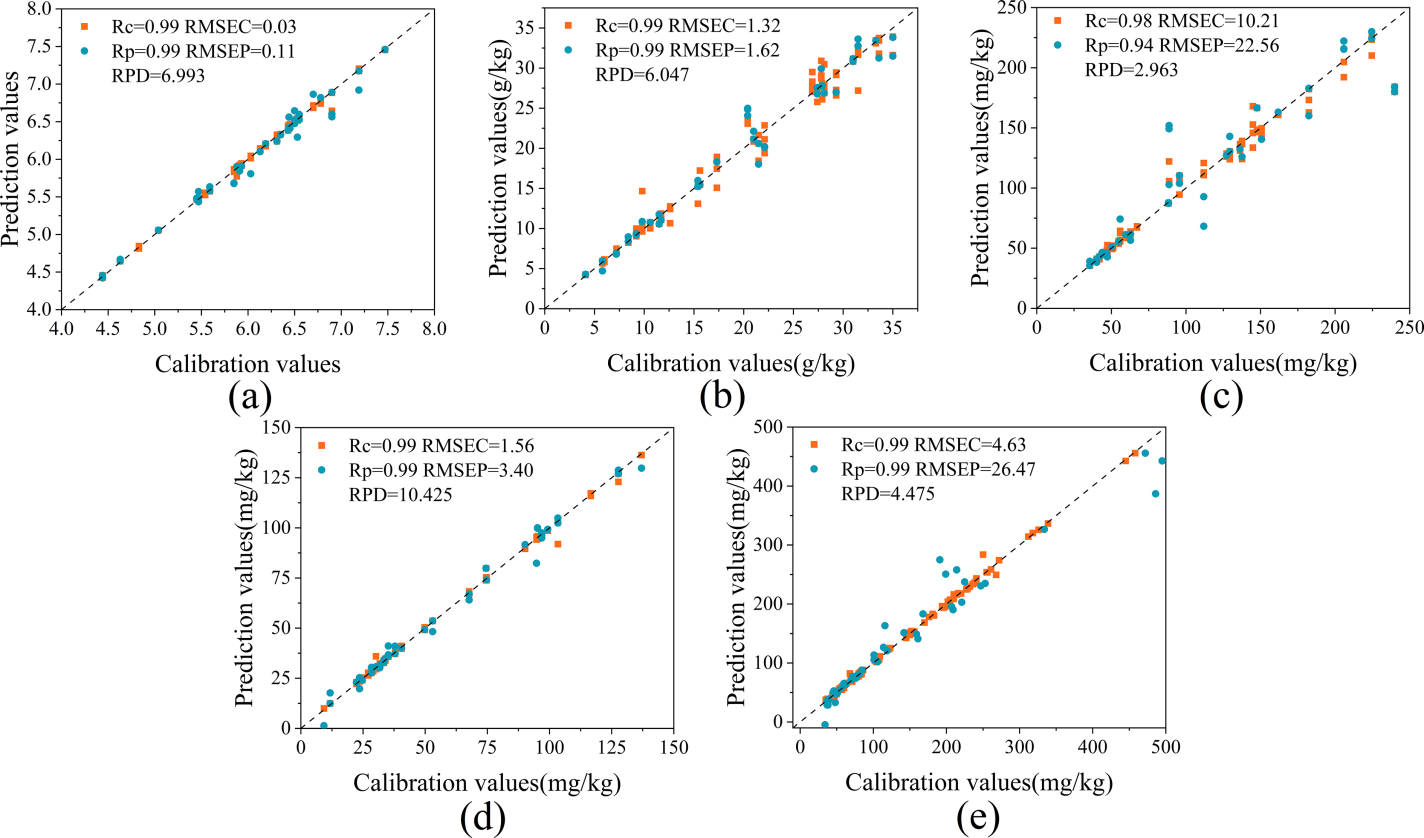
Scatter plots of measured versus predicted values for the optimal hyperspectral prediction models of soil physicochemical properties. **(a)** pH (Medfilt + BOSS + SVR); **(b)** SOM (Medfilt + BOSS + SVR); **(c)** AN (Normalize + BOSS + SVR); **(d)** AP (Medfilt + BOSS + SVR); **(e)** AK (Medfilt + BOSS + SVR).

Owing to the complex non-linear relationships between hyperspectral data and soil physicochemical properties, a single linear model struggles to adequately characterise the mapping relationship between spectral variables and target parameters. Therefore, building upon the construction of partial least squares regression (PLSR) models, two non-linear models—random forest (RF) and support vector regression (SVR)—were introduced for model optimisation and selection. The model evaluation metrics are presented in **Table 4**. For the prediction of soil pH and organic matter (SOM), the SVR models constructed under the combined Medfilt + BOSS condition both demonstrated optimal performance. The Rc and Rp of the pH prediction model both reached 0.99, with an RMSEP of 0.11 and an RPD of 6.993. Similarly, the Rc and Rp of the SOM prediction model were both 0.99, with an RMSEP of 1.62 and an RPD of 6.047; both exhibited high predictive accuracy and stability. In the prediction of available hydrolysable nitrogen (AN), the SVR model based on the normalize + BOSS combination demonstrated robust predictive capacity, with an Rc of 0.98, an Rp of 0.94, an RMSEP of 22.56, and an RPD of 2.963. Although the Rp of the RF model was slightly higher, the SVR model possessed a lower prediction error and a higher RPD value, resulting in superior comprehensive predictive performance. For the prediction of available phosphorus (AP), the Medfilt + BOSS + SVR model was the most outstanding; its Rc and Rp both reached 0.99, with an RMSEP of 3.40 and an RPD of 10.425, indicating exceptionally strong predictive capacity. For available potassium (AK), the SVR model likewise achieved the best results (Rc = 0.99, Rp = 0.98, RMSEP = 26.47, RPD = 4.475), and its predictive performance was markedly superior to that of the PLSR and RF models. Scatter plots for the optimal model combinations of each physicochemical component are shown in **Figure 7**. A comprehensive comparison of the predictive effects of the different models reveals that the SVR model exhibited superior stability and accuracy in predicting the majority of the soil physicochemical parameters (pH, SOM, AN, AP, and AK), indicating its distinct advantage in handling the non-linear relationships inherent in hyperspectral data. Concurrently, the BOSS variable selection method, combined with appropriate spectral pre-processing, can effectively improve the predictive accuracy of the models, providing reliable technical support for the rapid inversion of soil nutrient information.

**Table 4:** Performance comparison of different models for predicting soil physicochemical properties based on optimal spectral preprocessing and BOSS variable selection.

Properties	Optimal Combination	Model	PCs	Rc	RMSEC	Rp	RMSEP	RPD
pH	Medfilt+BOSS	PLSR	13	0.82	0.40	0.90	0.35	1.858
		RF	13	0.99	0.21	0.95	0.33	2.472
		SVR	13	0.99	0.03	0.99	0.11	6.993
SOM	Medfilt+BOSS	PLSR	11	0.95	3.02	0.96	2.84	3.405
		RF	11	0.99	2.14	0.98	2.92	2.531
		SVR	11	0.99	1.32	0.99	1.62	6.047
AN	Normalize+BOSS	PLSR	11	0.83	33.27	0.89	26.15	1.938
		RF	11	0.99	16.70	0.95	24.80	2.735
		SVR	11	0.98	10.21	0.94	22.56	2.963
AP	Medfilt+BOSS	PLSR	10	0.81	21.69	0.78	22.47	1.424
		RF	10	0.99	8.71	0.97	3.40	1.953
		SVR	10	0.99	1.56	0.99	3.40	10.425
AK	Medfilt+BOSS	PLSR	10	0.77	76.44	0.86	50.54	1.870
		RF	10	0.99	27.37	0.97	48.09	2.941
		SVR	10	0.99	4.63	0.98	26.47	4.475

### 3.6 Accuracy analysis of the optimal models for soil physicochemical components

**Figure 8:**
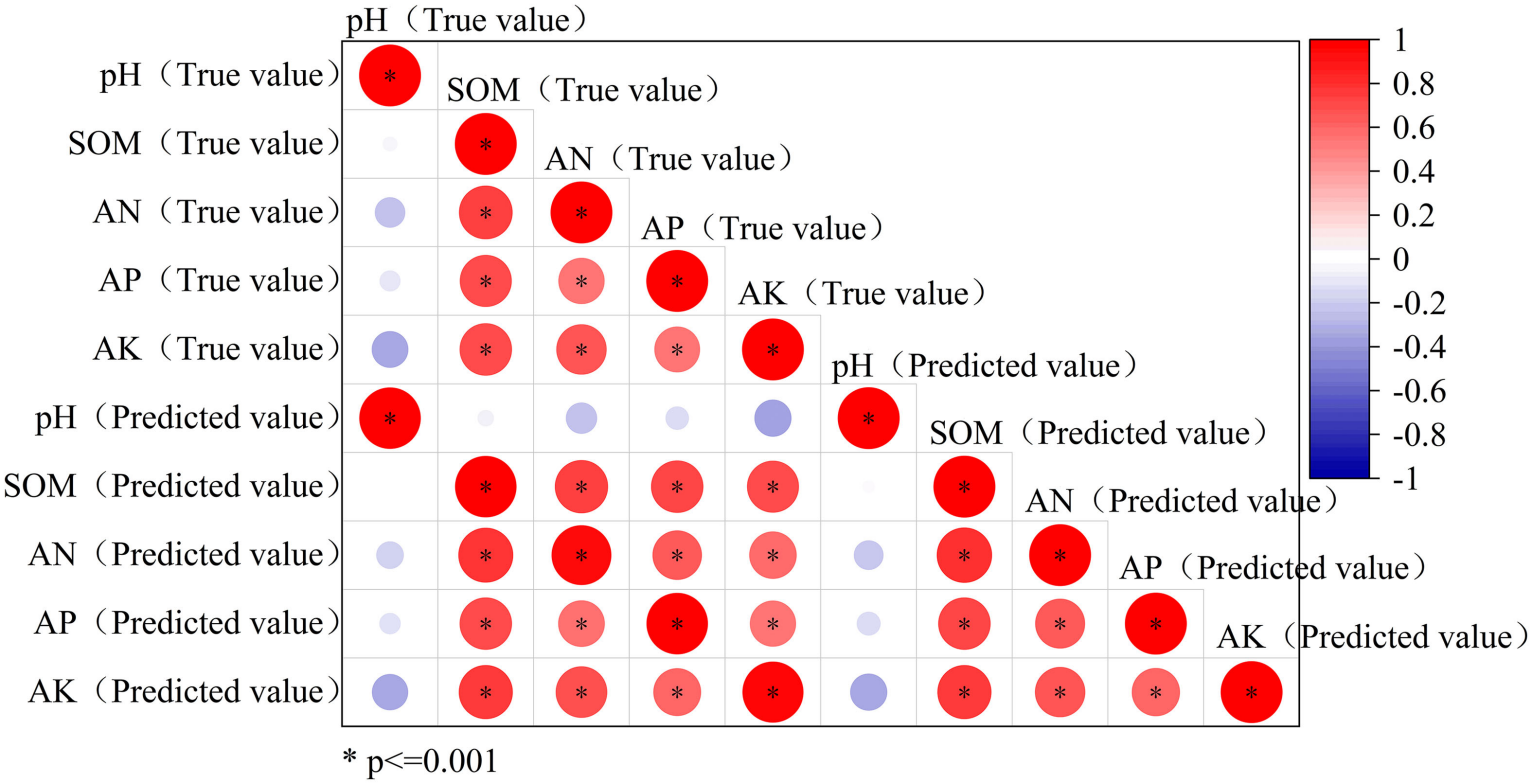
Correlation heatmap between observed and predicted values of soil physicochemical properties.

To facilitate the subsequent development of in-field non-destructive testing equipment for soil physicochemical components in tea gardens, the predictive capacity of the optimal models for each parameter was further evaluated. In addition to traditional model evaluation metrics, Pearson’s r and P-values were employed to assess the correlation between the measured and predicted values (**Table 5**). Highly significant correlations (P< 0.001) were observed between the measured and predicted values for soil pH and the four physicochemical parameters (SOM, AN, AP, and AK), demonstrating the exceptional performance of the established models. As illustrated in **Figure 8**, a correlation heatmap was generated for the measured and predicted values of the soil physicochemical components, revealing a highly significant positive correlation across all parameters.

**Table 5:** Pearson correlation coefficient and p-value between the observed and predicted values of soil physicochemical properties based on the optimal model.

Properties	Pearson r	P-value
pH	0.99	<0.001
SOM	0.99	<0.001
AN	0.95	<0.001
AP	0.99	<0.001
AK	0.97	<0.001

### 3.7 Visualisation results of soil physicochemical component contents

Compared with traditional spectroscopy, hyperspectral technology offers the dual advantages of spatial image information and spectral characteristics, enabling the spatial visual inversion of soil physicochemical properties. Building upon the aforementioned research foundation, the soil hyperspectral images were subjected to initial processing (importation and band cropping), optimal spectral pre-processing, optimal band selection, and principal component analysis (PCA) for dimensionality reduction. Finally, these data were inputted into the optimal SVR models to achieve point-by-point prediction of soil pH and physicochemical component contents (SOM, AN, AP, and AK) for each pixel. Concurrently, to avoid interference from background noise, a threshold mask based on average pixel reflectance was constructed to eliminate invalid regions. Ultimately, the valid predicted values were reconstructed into a two-dimensional spatial distribution map and mapped back onto the original soil hyperspectral images.

**Figure 9:**
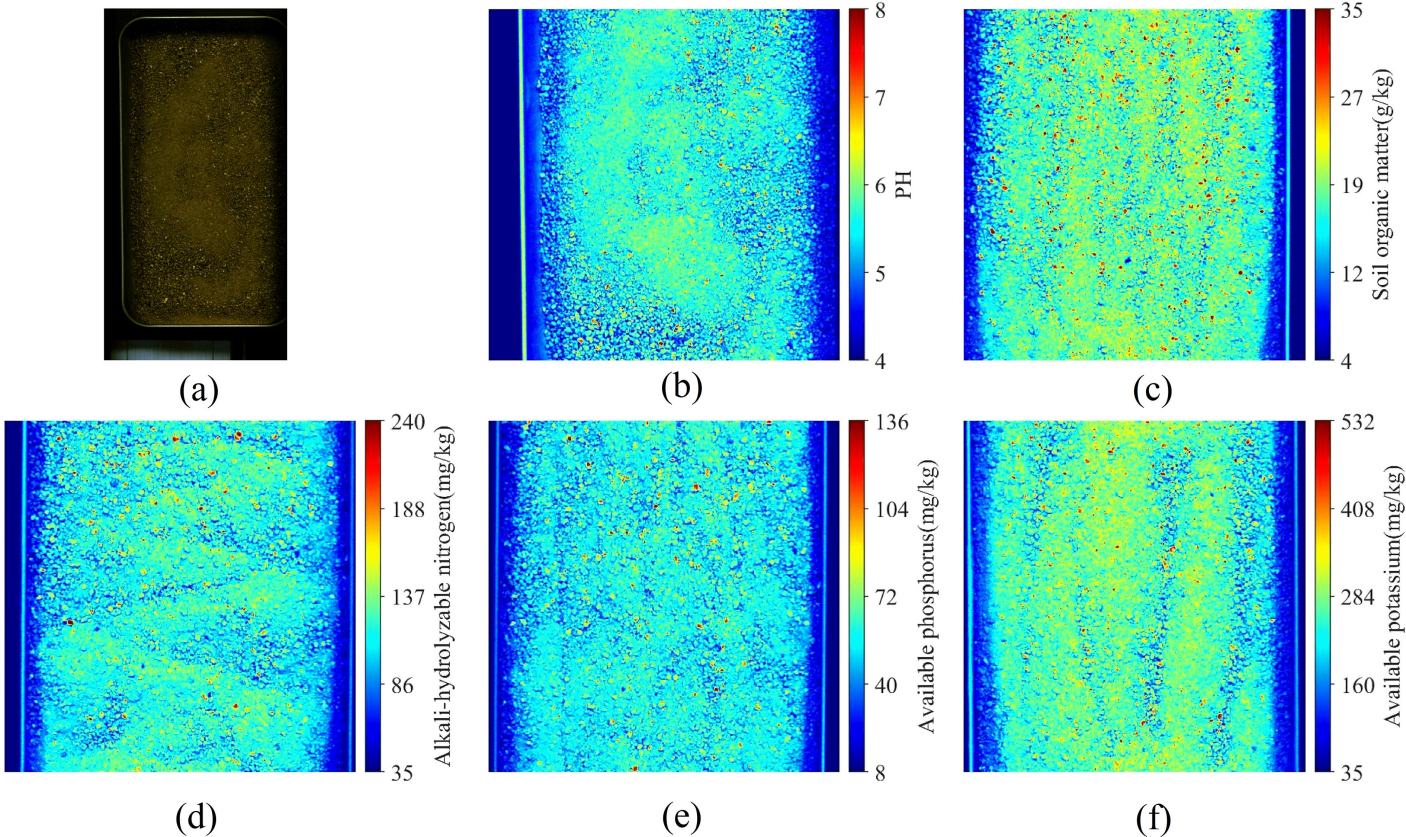
Visualization of soil pH and physicochemical properties derived from hyperspectral inversion. **(a)** Original hyperspectral image; **(b)** pH; **(c)** Soil organic matter (SOM); **(d)** Alkali-hydrolyzable nitrogen (AN); **(e)** Available phosphorus (AP); **(f)** Available potassium (AK).

During the visualisation stage, to preserve the true physical dimensions of the predicted values and effectively suppress the interference of extreme outliers on the visual presentation, this study utilised the 2% to 98% percentile range of the pixel predicted values for adaptive contrast stretching. This was combined with a perceptually uniform Turbo pseudo-colour mapping (with colours transitioning gradually from blue to red, representing the spatial variation of soil property contents from low to high) to generate the final spatial distribution maps (**Figure 9**). The overall results indicate that the differently coloured regions in the visualised images can clearly reflect the spatial heterogeneity of the soil physicochemical properties: low-value regions predominantly appear blue or cyan, moderate-level regions present as green to yellow, whilst high-value regions gradually transition to orange or even red. The visualisation method based on hyperspectral inversion can intuitively reveal the spatial distribution patterns of soil properties, providing a crucial basis for precision agricultural management and soil quality evaluation.

## 4 Discussion

Our findings reveal a pronounced vertical gradient in soil nutrients, characterized by surface enrichment and a sharp decline with depth. This pattern is closely linked to the “southern tea introduced to the north” management practices, where long-term fertilization and litter decomposition primarily affect the top 0–20 cm layer (Wu et al., 2022). The PCA results (**Figure 2**) and correlation analysis (**Figure 3**) further elucidate the coupled dynamics of soil properties. The high positive correlation between SOM and nitrogen/phosphorus suggests that organic matter acts as a primary reservoir and driver for nutrient cycling in these acidic soils. Interestingly, soil pH exhibited a relatively independent variation, likely influenced by the parent material and leaching processes specific to the Mount Tai region. The successful spatial visualization of these gradients (**Figure 8**) provides a powerful tool for monitoring soil degradation and nutrient stratification.

The core innovation of this study lies in the development of a high-precision retrieval framework specifically tailored for high-latitude tea plantation soils. Unlike previous studies that predominantly focused on topsoil in southern China, our approach integrates advanced feature selection (BOSS) with non-linear support vector regression (SVR) to capture the complex spectral responses of multiple soil properties across a 0–60 cm profile. The BOSS algorithm demonstrated significant advantages in suppressing spectral redundancy by assigning weights through bootstrap resampling, which effectively identified the most informative bands related to the overtones of O-H and C-H groups. Our results show that the BOSS-SVR architecture consistently outperformed traditional PLSR models, particularly for AP and SOM, achieving RPD values exceeding 6.0. This suggests that the non-linear kernel-based approach is superior in resolving the overlapping absorption features and scattering effects inherent in soil matrices. This research has significant implications for the sustainable development of high-latitude tea industries. The developed hyperspectral framework allows for the rapid, non-destructive assessment of soil fertility without the need for laborious chemical analysis. By generating pixel-wise spatial distribution maps, tea plantation managers can pinpoint areas of nutrient deficiency or soil acidification with high precision. This “digital mapping” capability supports the implementation of variable-rate fertilization (VRF) strategies, which can optimize nutrient use efficiency, reduce environmental runoff, and ultimately improve the quality of Taishan tea.

Despite the high predictive accuracy achieved, this study has limitations. First, the hyperspectral data were acquired in a controlled laboratory environment using air-dried, sieved samples. Future research should explore the impact of soil moisture and surface roughness on *in-situ* field measurements to enhance the model’s practical applicability. Second, the spectral range used (394–1007 nm) focuses on the visible and near-infrared regions; incorporating the short-wave infrared (SWIR, 1100–2500 nm) might further improve the detection of specific mineral components and clay content. Lastly, while the current model is highly effective for the Taishan region, its transferability to other high-latitude tea regions with different soil parent materials remains to be tested. Future work will focus on cross-regional calibration transfer and the integration of multi-source remote sensing data for large-scale ecological monitoring.

## 5 Conclusion

This study has successfully achieved the development of high-precision prediction models and the spatial visual inversion of soil physicochemical properties (pH, SOM, AN, AP, and AK) in tea gardens, based on hyperspectral imaging and machine learning. The principal conclusions of this research are as follows:

Revealed the mechanisms of vertical differentiation and spectral responses of soil nutrients in tea gardens. The tea garden soils were predominantly acidic overall. The various physicochemical parameters (SOM, AN, AP, and AK) exhibited pronounced surface enrichment and distinct vertical gradient distribution characteristics along the soil profile. Concurrently, a clear synergistic variation (a significant positive correlation) was observed between SOM and nutrient indicators such as nitrogen, phosphorus, and potassium, whilst pH exhibited relatively independent variation characteristics.

Established optimal strategies for soil hyperspectral pre-processing and characteristic band selection, and constructed highly robust non-linear nutrient prediction models. Tailored to the various physicochemical parameters, median filtering (Medfilt) or normalize pre-processing, combined with the bootstrapping soft shrinkage (BOSS) algorithm, most effectively eliminated spectral redundancy and enhanced the performance of the prediction models. When addressing the complex non-linear mapping relationships between the hyperspectral data and soil physicochemical properties, support vector regression (SVR) demonstrated an absolute advantage. The correlation coefficients of the prediction set (Rp) for all physicochemical parameters exceeded 0.94. Notably, the predictive performance for available phosphorus (AP) was exceptionally outstanding (achieving an RPD of 10.425), thereby realising the high-precision quantitative prediction of soil physicochemical component contents.

Realised the spatial visual reconstruction of soil physicochemical component contents within complex habitats. Leveraging the hyperspectral advantage of integrating spatial and spectral information, this study inputted the soil hyperspectral images into the optimal combination models for each physicochemical parameter to conduct pixel-by-pixel prediction. Through background mask extraction combined with adaptive percentile stretching (2%–98%) and a perceptually uniform Turbo colour map, the point-based predicted values were successfully transformed into two-dimensional spatial distribution maps. This visualisation method effectively overcomes the spatial limitations inherent in traditional point-based analyses and intuitively reveals the spatial distribution of soil physicochemical properties. Consequently, it provides a reliable theoretical foundation and technical paradigm for precision nutrient management, digital agricultural development, and the dynamic assessment of ecological quality in the Taishan tea region, and by extension, high-latitude tea gardens across China.

## Data Availability

The original contributions presented in the study are included in the article/supplementary material. Further inquiries can be directed to the corresponding authors.
